# Epigenetic plasticity: A central regulator of epithelial-to-mesenchymal transition in cancer

**DOI:** 10.18632/oncotarget.1875

**Published:** 2014-03-28

**Authors:** Upasana Bedi, Vivek Kumar Mishra, David Wasilewski, Christina Scheel, Steven A. Johnsen

**Affiliations:** ^1^ Department of Molecular Oncology, Göttingen Center for Molecular Biosciences, University Medical Center Göttingen, Göttingen, Germany; ^2^ Department of Tumor Biology, University Medical Center Hamburg-Eppendorf (UKE), Hamburg, Germany; ^3^ Department of General, Visceral and Pediatric Surgery, University Medical Center Göttingen, Göttingen, Germany; ^4^ Institute of Stem Cell Research, Helmholtz Centre Munich, Munich, Germany

**Keywords:** Epigenetics, Chromatin, Cancer, Epithelial-to-mesenchymal transition, Metastasis

## Abstract

Tumor metastasis is the major cause of mortality and morbidity in most solid cancers. A growing body of evidence suggests that the epithelial-to-mesenchymal transition (EMT) plays a central role during tumor metastasis and frequently imparts a stem cell-like phenotype and therapeutic resistance to tumor cells. The induction of EMT is accompanied by a dynamic reprogramming of the epigenome involving changes in DNA methylation and several post-translational histone modifications. These changes in turn promote the expression of mesenchymal genes or repress those associated with an epithelial phenotype. Importantly, in order for metastatic colonization and the formation of macrometastases to occur, tumor cells frequently undergo a reversal of EMT referred to as the mesenchymal-to-epithelial transition (MET). Thus, a high degree of epigenetic plasticity is required in order to induce and reverse EMT during tumor progression. In this review, we describe various epigenetic regulatory mechanisms employed by tumor cells during EMT and elaborate on the importance of the histone code in controlling both the expression and activity of EMT-associated transcription factors. We propose that a more thorough understanding of the epigenetic mechanisms controlling EMT may provide new opportunities which may be harnessed for improved and individualized cancer therapy based on defined molecular mechanisms.

## INTRODUCTION

**Figure 1:**
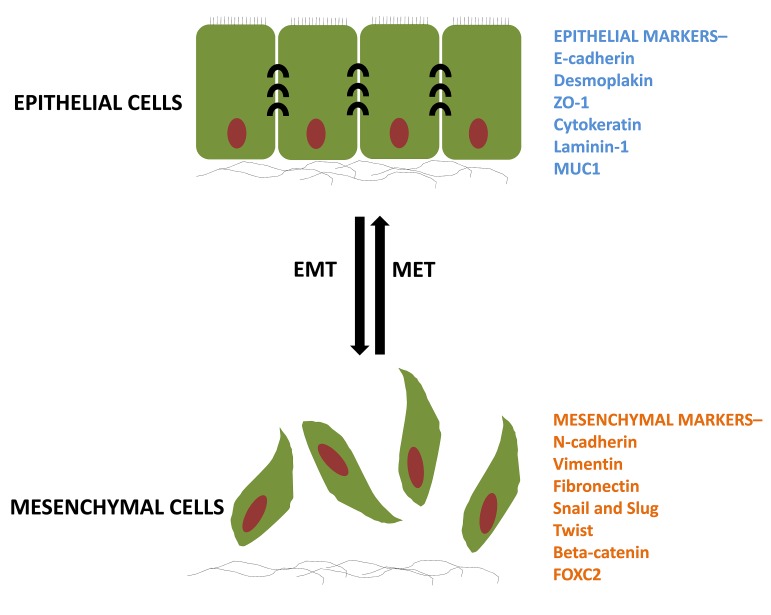
The process of EMT and its reversible MET Epithelial cells having a particular set of markers undergo biochemical changes and acquire different set of markers for a mesenchymal phenotype. ZO-1, zona occludens; MUC1, mucin 1; FOXC2, forkhead box C2.

Metastatic disease accounts for more than 90% of deaths in patients with solid tumors [1]. Metastasis is a complex process requiring tumor cells to invade into the surrounding tissue, gain access to vasculature, survive transport, exit the vasculature and then resume growth in a foreign tissue environment [2]. Our understanding of metastasis has been greatly improved by the recognition that cancer cells can acquire the ability to accomplish several steps of the metastatic process at once through the engagement of a latent cellular program, the Epithelial-Mesenchymal Transition (EMT) [3,4]. EMT plays an important role in controlling critical morphogenetic steps during normal embryonic developmental processes, and has been linked to the acquisition of cancer cell motility and invasiveness in solid malignancies. During both normal development and tumor progression, EMT is orchestrated by a set of pleiotropically acting transcription factors (TFs), such as Twist, Snail, Slug, ZEB1/2 that together form an intricate transcriptional circuitry [5–7]. Through the action of EMT-TFs, which mainly act as transcriptional repressors, cells lose epithelial traits, such as expression of E-cadherin and ZO-1, leading to the dissolution of adherens and tight junctions (Figure 1). Repression of epithelial markers is paralleled by transcriptional upregulation of mesenchymal adhesion molecules, such as N-cadherin and fibronectin and matrix degrading enzymes, respectively. Together, these changes enable epithelial cells to switch from an apical-basal polarity and restricted lateral, collective movement confined by the basement membrane to a front-to-back polarity with the ability to freely migrate and invade as single cells. Thus, in embryonic development, EMT is critical for mesoderm formation during gastrulation. Overall, EMT affects cellular distribution throughout the embryo during processes such as gastrulation and neural crest migration. Over the last few years EMT has emerged as a key process employed by cancer cells to accomplish the early steps of the metastatic process, including local tissue invasion, entry into blood and/or lymphatic vessels, survival during transit and exit from the circulation into distant tissue parenchyma.

Activation of an EMT program is also coupled with the ability of cancer cells to initiate experimental tumors in mice with high efficiency, although the exact molecular mechanisms linking EMT and tumor-initiating capacity of cancer cells still remain to be determined [8,9]. Given the similarity of experimental tumor initiation and establishment of macroscopic metastases, it is conceivable that EMT is involved in both the early and late steps of the metastatic cascade, which encompass outgrowth of disseminated tumor cells. Intriguingly, recent research points to the necessity of the reversal of EMT by means of a Mesenchymal-Epithelial Transition (MET) at the metastatic site to enable the outgrowth of disseminated tumor cells (DTC) into macroscopic metastases [10], supported by earlier work pointing to the importance of MET in the metastatic cascade [11]. These experimental studies are supported by the clinico-pathological observation that most metastases arising from carcinomas display an epithelial phenotype mimicking the differentiation patterns found in the cognate primary tumor site of a given tumor [12]. However, these seemingly opposing observations may be reconciled by comprehending EMT as a highly dynamic and reversible process. In this scenario, the most aggressive tumor cells would be predicted to be those displaying a high degree of cellular plasticity or a mixed phenotype integrating epithelial and mesenchymal characteristics. This model is supported by recent observations of dynamic changes in epithelial and mesenchymal features in circulating tumor cells of breast cancer patients [13]. Together, these data suggest that inhibition of epithelial plasticity is an attractive approach for therapeutic intervention aimed at inhibiting cell-state transitions, rather than targeting mutated or otherwise genetically altered gene products. However, the precise molecular links between EMT programs and cellular plasticity are just beginning to be unraveled.

Considering the dynamic and drastic transcriptional changes that occur during both EMT and MET, broad sweeping, reversible changes in epigenetic modifications which affect chromatin state, represent a central and crucial regulatory component of the metastatic process. Changes in gene expression do not depend solely upon the availability of appropriate transcription factors, but also upon the degree of “openness” or “closedness” of the chromatin since both the binding of a TF to DNA as well as its ability to recruit additional transcriptional co-regulatory proteins depends upon changes in histone modifications at the target gene. In this regard, emerging data have shown that EMT also involves epigenetic reprogramming with widespread alterations to chromatin modifications at both the DNA and protein level. For example, EMT-TFs, such as Twist, Snail, Slug, ZEB1 and ZEB2 recruit various histone-modifying complexes to chromatin, thereby mediating epigenetic silencing of genes [14]. Here, we review the interplay between EMT-TFs, transcriptional regulation of EMT markers and chromatin modifiers. Emphasis is laid primarily on histone modifications largely due to their amenability to intervention in possible future therapies to prevent metastasis or metastatic relapse.

### Epigenetic Control

Epigenetic regulation of gene expression occurs largely through reversible chemical modification of DNA or histone proteins, which do not alter the DNA sequence, but instead control its accessibility and/or ability to be read [15]. Certain epigenetic modifications are almost exclusively associated with constitutively silenced regions of the genome (“heterochromatin”) while other modifications are almost exclusively found in actively transcribed regions of the genome (“euchromatin”) [16]. Other epigenetic changes in chromatin structure can occur through the exchange of variant histones or assembly and disassembly of chromatin structure via histone chaperones, or through ATP-dependent chromatin remodeling, for example, by members of the Swi/Snf family of proteins [17,18]. Substantial changes in epigenetic modifications occur to different degrees during various developmental processes such as germ cell development and stem cell differentiation [19], as well as during pathologic processes such as tumorigenesis [20]. Chromatin is composed of DNA wrapped around a nucleosome containing two of each of the core histones H2A, H2B, H3 and H4. Each of the core histone proteins can be modified at the post-translational level in various ways including the acetylation, methylation, ubiquitination and sumoylation of the amino terminus of lysine side chains; methylation or citrullination of arginine residues; as well as phosphorylation of serine, threonine and tyrosine residues [21,22]. The specific combination of histone modifications, commonly referred to as the “histone code”, is thought to determine the functional outcome, probably largely due to the recruitment of scaffolding proteins such as bromo- and chromodomain proteins which specifically recognize acetylated and methylated lysine residues, respectively [21,23]. A major emphasis has been placed on understanding the role of DNA methylation in cancer, but more studies are beginning to determine the roles of various histone modifications, their modifying enzymes and overall chromatin structure in controlling tumor progression and metastasis. This has been fueled at least in part by the recent discovery that epigenetic regulatory proteins are the frequent targets of genetic mutation in many types of hematological malignancies and solid tumors [24]. However, the role of most of these mutations in EMT or metastasis remains unknown.

Several signaling networks including hypoxia, TGFβ, Wnt and Notch signaling may all activate EMT by wide-spread transcriptional changes via the activation of specific transcription factors [25] which elicit their effects on gene transcription and the epigenetic landscape by recruiting epigenetic regulatory proteins to specific genes, including those associated with an epithelial or mesenchymal phenotype. Therefore, a better understanding of the functional interaction of epigenetic modifiers with EMT-TFs, their specificity in the EMT and MET processes as well as their specificity for certain tumor types may lead to the identification of new therapeutic targets for preventing metastasis or metastatic outgrowth.

### Epigenetic Writers

Epigenetic modifiers can be largely classified into the categories of “epigenetic writers”, “epigenetic readers” and “epigenetic erasers”. Proteins that catalyze a specific histone modification are referred to as “epigenetic writers”. Examples of epigenetic writers include DNA and histone/lysine methyltransferases (HMT/KMT), histone/lysine acetyltransferases (HAT/KAT), arginine methyltransferases (PRMT), ubiquitin ligases, etc. As indicated, the EMT transcriptional program is controlled both by DNA methylation [26] and post-translational histone modifications [27].

In mammalian cells, DNA is methylated at the cytosine residues of CpG dinucleotides and is commonly associated with gene repression and heterochromatin formation [28]. In cancer cells, the genome is globally hypomethylated whereas CpG islands are frequently hypermethylated, resulting in reduced expression of tumor suppressor genes [29]. For example, the *CDH1* gene encoding E-cadherin is frequently hypermethylated in breast cancer cell lines exhibiting an EMT-like phenotype [30] and is also shown to be methylated along with several other genes silenced in basal-like breast cancers [31]. In addition to methylation of cytosine, subsequent hydroxylation of 5mC to 5-hydroxymethyl-cytosine (5hmC) and further oxidation to non-methylated cytosine by the Ten-Eleven Translocation (TET) family of methylcytosine dioxygenases plays a tumor suppressor function in many types of cancers [32,33,34]. Notably, TET1 is frequently down-regulated in breast and prostate cancer in cell lines and xenograft models and its downregulation is associated with overall poorer patient survival [32]. This effect appears to be at least partially due to a TET1-dependent demethylation and activation of the Tissue Inhibitor of Metalloproteinase (TIMP)-2 and 3 genes, which are established suppressors of the EMT phenotype. Thus, alterations in both 5mC and 5hmC caused by mutation or repression of the TET proteins may play a role in promoting EMT in solid malignancies. Recently, a role for the TET proteins in metastasis has been shown by demonstrating that the microRNA-22 (miR-22) exerts its prometastatic effects by directly targeting TET proteins [35]. In this way, downregulation of the TET proteins prevents demethylation of the miR-200 gene, which targets the mRNAs for established regulators of the EMT program such as the EMT-TF ZEB1, TGFβ1 and the polycomb protein BMI1, thereby potently antagonizing activation of an EMT program and metastasis.

*Histone Acetyltransferases (HATs)* – An important and well-studied modification responsible for making chromatin accessible to transcription factors is histone acetylation [36]. Histone acetyltransferases (also referred to as lysine acetyltransferases, KATs) such as GCN5 (KAT2A), P/CAF (KAT2B), p300 (KAT3B) and CBP (KAT3A) transfer acetyl groups to the amino group of lysine side chains of histones, thereby altering the charge of the histone, relaxing the chromatin and making it more accessible to transcription factors [37]. One important HAT, p300, affects the regulation of Snail and ZEB1 in colon cancer, thereby contributing to EMT and tumor progression [38]. A different study reported that the absence of p300 promotes EMT in the HCT116 colorectal cancer cell line [39]. Other HATs such as the human homolog of *males absent on the first* (hMOF/KAT8) as well as the Steroid Receptor Coactivators-1 and -3 (SRC1/NCOA1 and SRC3/NCOA3) have been shown to play tumor and metastasis suppressor and activator roles, respectively [40,41,42,43,44]. However, future studies are needed to address whether and how these or other HATs regulate EMT.

*Histone Methyltransferases (HMTs)* – Methyltransferases transfer methyl groups to the lysine or arginine residues of histones. They are classified into lysine (KMT) or arginine methyltransferases (PRMT) depending on the substrate residue for methylation. SET (*Su(var) 3-9*, *Enhancer of Zeste* and *Trithorax*) domain containing enzymes such as G9a, SUV39H1/H2, EZH1/2 and others, transfer one to three methyl groups to lysine residues on histones [45,14]. Expressed genes typically display “active” methylation marks such as H3K4me3, H3K36me3 and H3K79me3, while transcriptionally silenced genes generally exhibit “repressive” marks such as H3K27me3, H3K9me2 and H3K9me3. Upon hypoxia, mesenchymal genes are marked with H3K4me3 by WDR5, part of MLL and SET1 HMT complex [46]. The Polycomb Repressor Complex-2 (PRC2), which contains the methyltransferase Enhancer of Zeste Homolog-2 (EZH2) in complex with Suppressor of Zeste-12 (SUZ12) and Embryonic Ectoderm Development (EED) [47], plays a key role in transcriptional silencing by mediating H3K27me3 [48]. The role of PRC2 in tumorigenesis and EMT has been well characterized and its interplay with EMT-TFs is described in more detail below.

*Histone Ubiquitin Ligases* – Ubiquitination involves the attachment of one or more 76 amino acid ubiquitin moieties to the side chain of a lysine in a process involving the sequential function of three enzymes: E1 ubiquitin-activating, E2 ubiquitin-conjugating and E3 ubiquitin-ligase enzymes [49]. While polyubiquitination via lysine 48 of ubiquitin frequently targets proteins for degradation via the 26S proteasome, monoubiquitination does not usually target proteins for degradation, but rather functions like other post-translational modifications to serve as a mark for recognition by other proteins or directly alter protein structure or function. In the case of chromatin, both histones H2A and H2B can be monoubiquitinated in mammals at Lys-119 (H2Aub1) or Lys-120 (H2Bub1), respectively. H2Bub1 is generally associated with euchromatin and transcriptional elongation [50,51,52], whereas H2Aub1 is localized to regions of heterochromatin and prevents transcriptional elongation [53,54]. H2B is monoubiquitinated by the obligate RNF20/40 heterodimer in a complex with the ubiquitin conjugating enzyme UBE2A (human homolog of yeast Rad6A) [55]. While decreased H2Bub1 levels are associated with increased invasiveness and tumor progression, its role in controlling EMT has not been described yet [56,57,58,89]. In contrast, components of the Polycomb Repressor Complex-1 (PRC1), which ubiquitinates H2A have been shown to promote EMT by upregulating Snail via modulation of PI3K/Akt/GSK-3β signaling [59] as well as targeting other important EMT transcription factors such as Twist1 and ZEB1 [60,61].

### Epigenetic Readers

Once the chromatin has been marked with specific post-translational histone modifications, the regulatory output in most cases is achieved by the recognition of those marks by epigenetic readers. These chromatin regulators possess specialized domains that recognize and bind to various histone modifications and control DNA-associated functions by recruiting additional regulatory proteins and/or by directly affecting chromatin structure [62].

*Bromodomains* – Bromodomain-containing proteins recognize acetylated lysine residues [63,64]. One particularly noteworthy subclass of bromodomain proteins is the BET (Bromodomain and Extra Terminal) family of proteins which contain two tandem bromodomains and an Extraterminal domain (ET) [65]. This family consists of BRD2, 3, and 4 as well as the testis-specific BRDT protein and is implicated in transcriptional regulation by binding to chromatin via the bromodomains [66]. In addition to its established role in promoting leukemiogenesis by MLL translocation products [67] and its fusion with NUT in NUT midline carcinoma [68,69,70], BRD4 was shown to suppress an EMT phenotype in mammary epithelial cells [71]. However, additional studies are necessary to further characterize the function and molecular mechanisms of BRD4 and other BET domain proteins in EMT during tumor progression and metastasis. Notably BET domain protein function can be inhibited by small molecule inhibitors such as JQ1 which specifically binds to the bromodomains within BET proteins such as BRD4 and prevents binding to acetylated chromatin [72]. Inhibition of BRD4 has been shown to have a positive effect on MYC-dependent tumor cells [73,74]. However, given a potential EMT suppressor function of BRD4, the potential effects of JQ1 treatment on breast cancer cell phenotype need to be clarified.

*Recognition of Methylated Lysine Residues* – Analogous to the recognition of acetylated lysine residues by bromodomains, a number of different domains have been identified which recognize methylated lysine residues including Chromatin organization modifier (chromo-), TUDOR, Plant Homeodomain (PHD) and Malignant Brain Tumor (MBT) domains [75]. SFMBT1, a MBT domain containing protein, whose expression has been associated with bad prognosis, forms a complex with the demethylase LSD1 and is recruited to epithelial genes via interaction with SNAI1 to promote gene repression by demethylating H3K4me2 [76]. Within the chromodomain family of proteins there are three sub-families: the heterochromatin protein (HP1)/chromobox (CBX) proteins, the chromodomain helicase DNA binding domain (CHD) subfamily and the chromo barrel domain family [77]. CBX proteins are components of the PRC1 complex which recognizes H3K27me3 to promote H2Aub1 and transcriptional repression at PRC2 targets [78,79]. CBX4 mediates sumoylation of Smad-interacting protein 1 (SIP1), which along with ZEB2, is involved in *CDH1* repression and EMT [80,81]. Another member, MPP8 (M-phase phosphoprotein 8) recognizes H3K9 methylation on chromatin and interacts with HMTases GLP and ESET as well as the DNA methyltransferase DNMT3A. MPP8 in turn functions to repress *CDH1* expression thereby promoting EMT [82].

**Figure 2:**
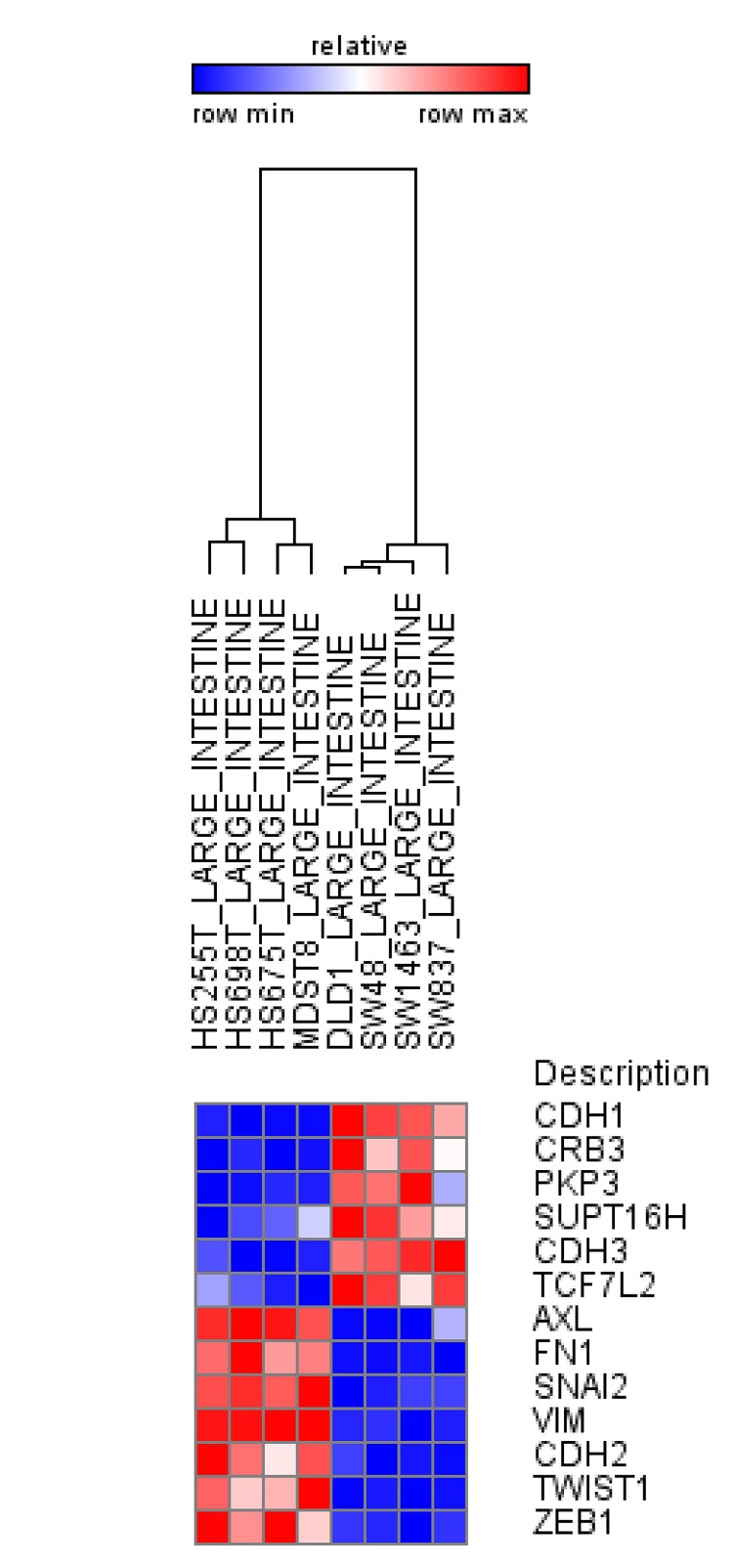
SUPT16H, FACT subunit correlates with the epithelial phenotype in human intestinal cell lines Analysis using data from Cancer Cell Line Encyclopedia indicates increased expression of epithelial markers for SUPT16H and decreased expression of mesenchymal markers in intestinal cell lines.

*Chromatin Remodeling Proteins and Histone Chaperones* – The regulation of chromatin organization and structure requires both the ATP-dependent activity of chromatin remodeling proteins as well as the ATP-independent functions of histone chaperones [83,84]. One component of the Swi/Snf family of ATP-dependent chromatin remodeling proteins BRG1 was found to be mutated in various human tumor cell lines [85] and appears to function with β-catenin at TCF target gene promoters to facilitate Wnt/β-catenin-regulated gene transcription in colon carcinoma cells [86]. Importantly, BRG1 also interacts directly with the EMT-TF ZEB1 to repress *CDH1* expression and promote EMT [87]. In contrast, Metastasis-associated gene 3 (MTA3), part of the ATP-dependent NuRD/Mi-2/CHD remodeling complex was shown to suppress EMT by directly repressing *SNAI1* expression [88]. Although the activity of histone chaperones has not yet been linked to EMT, our recent data identified decreased expression of the human *Suppressor of Ty Homologue-6* (SUPT6H) during breast cancer progression which was associated with decreased H2Bub1 levels, a loss of estrogen responsiveness and a shift from a luminal epithelial to myoepithelial phenotype [89]. Another histone chaperone complex referred to as Facilitates Chromatin Transcription (FACT) has been implicated in tumorigenesis [90] and DNA repair [91]. Interestingly, an analysis of gene expression data from the Cancer Cell Line Encyclopedia [92] suggest that higher expression of the FACT subunit, Suppressor of Ty Homolog 16 (SUPT16H) is more closely correlated with expression of the epithelial markers CDH1, CRB3, PKP3 and CDH3, and inversely correlated with the expression of the mesenchymal markers AXL, FN1, SNAI2, VIM, CDH2, TWIST1 and ZEB1 (Figure 2). Thus, whether and how FACT activity is correlated with an EMT phenotype may be of particular relevance for the application of molecules targeting FACT activity. Future studies will be necessary to determine whether and how SUPT6H, SSRP1, SUPT16H and other histone chaperones promote an EMT phenotype.

### Epigenetic Erasers

In contrast to the notion of epigenetic inheritance, most epigenetic modifications, including extremely stable modifications such as DNA methylation, are highly dynamic and can be added or removed from genes within minutes, frequently in a cyclic fashion [93,94,95]. After the initial activation or repression of a gene has been achieved, cellular and transcriptional plasticity is maintained by the reversibility of the epigenetic status of the target genes. In order to achieve this, most histone modifications also have specific enzymes that catalyze their removal. This class of proteins is broadly referred to as “epigenetic erasers” and exerts an equally important function as writers. If the signal is not stopped in a timely manner, the results can lead to defects in transcription and DNA repair ultimately promoting tumorigenesis of tumor progression [96].

*Histone Deacetylases (HDACs)* – The acetyl groups added by HATs are removed by HDACs in a highly regulated fashion which generally leads to chromatin compaction and transcriptional repression. Notably, HDAC1 was found to be important for TGFβ1-induced EMT [97] and its inhibition suppressed TGFβ1-induced EMT [98]. HDAC3 also interacts with WDR5, a core component of the histone methyltransferase complex responsible for H3K4 methylation and induced hypoxia-mediated EMT by regulating acetylation and methylation patterns on EMT genes [46]. Furthermore, the NAD^+^-dependent histone deacetylase SIRT1 was shown to cooperate with ZEB1 to silence *CDH1* expression by deacetylating its promoter [99].

*Histone Demethylases* – Finally, histone demethylases revert the effect of HMTs by removing the methylation marks on histones. The first histone demethylase identified was Lysine-Specific Demethylase-1 (LSD1 or KDM1A) [100], which removes mono- and di- methyl groups from H3K4. During EMT, SNAI1 recruits LSD1 to epithelial gene promoters and represses transcription by removal of dimethylation from H3K4 [101,102,103]. Two other demethylases belonging to the Jumonji-domain family, KDM6B (JMJD3) which removes H3K27me3 and KDM4B (JMJD2B), responsible for demethylation of H3K9me3 and H3K36me3, were recently shown to promote EMT as well [104,105].

*Histone Deubiquitinases (DUBs)* – As with essentially all other post-translational modifications, the ubiquitin moiety from histones can also be removed in order to reverse the effects of ubiquitination. One example is Ubiquitin-Specific Protease-22 (USP22) which deubiquitylates histone H2B [106] and was found to regulate BMI1-mediated INK4a/ARF and Akt signaling [107]. Consistently, USP22 is upregulated in tumors with a stem cell-like phenotype from patients exhibiting overall poorer survival [106,108,109]. Although many lines of research suggest that the positive and negative regulation of H2Bub1 could be associated with a tumor stem cell-like phenotype and EMT, further work will be needed to address this. The ubiquitination of H2A was reported to be reversed by a number of different DUBs including USP3 [110]. Notably, USP3 depletion induces scattering of A549 epithelial lung cancer cells, possibly reflecting a more mesenchymal cellular phenotype [111]. However, how and whether H2A deubiquitination is involved in controlling EMT still needs to be resolved.

### Epigenetic Regulation of EMT-Inducing Transcription Factors

**Figure 3:**
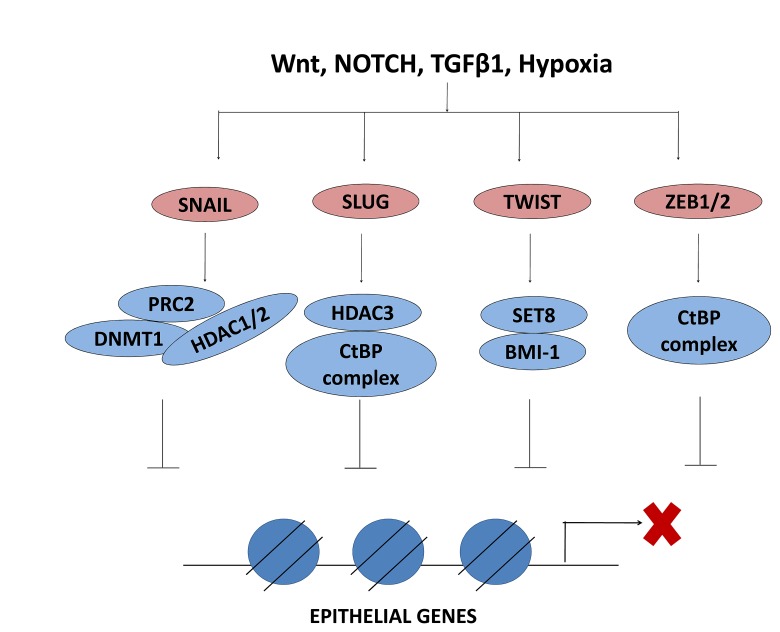
EMT-TFs interact with epigenetic regulators to repress epithelial genes EMT inducing factors activate the EMT-TFs which in turn interact with epigenetic regulators to repress the expression of epithelial genes. PRC2, polycomb repressive complex 2; DNMT1, DNA methyltransferase 1; HDAC, histone deacetylase; CtBP, C-terminal binding protein; BMI1, B lymphoma Mo-MLV insertion region 1; SET, Su(var) 3-9, Enhancer of Zeste and Trithorax

The cellular plasticity which allows the inter-conversion between epithelial and mesenchymal phenotypes via EMT and MET requires a complex network of interactions between different EMT-TFs, ubiquitous TFs and the epigenetic regulators described above. Herein, both the expression and the activity of EMT-TFs are controlled at an epigenetic level. The connection between loss of E-cadherin and tumor progression has been well established [112], and studies have highlighted the epigenetic regulation of the *CDH1* gene encoding E-cadherin to be instrumental for cancer cell metastasis [113]. *CDH1* expression is regulated by EMT-TFs including the Snail transcription factor family members Snail (SNAI1) and Slug (SNAI2) [114]. Research has shown that Snail recruits several chromatin modifying enzymes, such as LSD1, G9a, Suv39H1, HDAC1/2 and PRC2, to the *CDH1* promoter for transcriptional repression [115,116,117,118]. Figure 3 and Table 1 list the described interactions of EMT-inducing factors with various epigenetic factors to transcriptionally repress epithelial genes during EMT.

**Table 1:** Epigenetic factors involved in EMT

Transcription factor	Molecular function	Modifications	EMT regulation	Refs
HDACs	Histone deacetylases	H3K ac	WDR5 controls H3K4me3 on EMT genes; CDH1 repression with Snail & Twist1	[46,133,134]
EZH2	Polycomb protein complex 2 (PRC2) component, histone methyltransferase	H3K27me3	Activated by SOX4; EMT epithelial gene repression; often overexpressed	[121,135]
SUZ12	Polycomb protein complex 2 (PRC2) component	H3K27me3 with EZH2	Often overexpressed	[136]
BMI1	Polycomb protein complex 1 (PRC1) component; gene silencing	H2Aub1	Upregulated during EMT; EMT gene repression with Snail & Twist1	[137,138,139,140]
SUV39H1	Histone methyltransferase	H3K9me3	SNAI1 mediated CDH1 repression	[115]
G9a	Histone methyltransferase	H3K9me2	SNAI1 mediated CDH1 repression	[116]
LSD1	Histone demethylase	H3K4me1/2; H3K9me1/2	SNAI1 mediated repression	[100,141,142]
KDM6B (JMJD3)	Histone demethylase	H3K27me3	SNAI1 mediated repression	[104]

*SOX4 – An Important Upstream Regulator of the EMT Program –* SOX4 is a member of the Sox (SRY-related HMG-box) family of transcription factors and is frequently upregulated in various cancer types [120]. A recent study demonstrated that SOX4 acts early in the induction of the EMT pathway [121]. Upon TGFβ1 induction, SOX4 expression is increased, thereby transcriptionally activating *EZH2* expression, which in turn increases H3K27me3 at specific genes in order to promote EMT. In concordance with this, depletion of either the transcription factor SOX4 or its epigenetic regulatory partner EZH2 similarly prevented TGFβ-induced EMT in the murine mammary epithelial cell line NMuMG. Similarly, SOX4 overexpression induced EMT via modulation of Ezh2-mediated H3K27me3 marks on several EMT genes. Together these results strongly implicate SOX4 as a critical upstream regulator of the EMT modulators that carries out its function via epigenetic mechanisms involving EZH2.

SUV39H1 (*Suppressor of Variegation 3-9* Homolog 1), is a key HMT responsible for trimethylation of H3K9. H3K9me3, like H3K27me3, is a histone modification associated with gene repression. Recently, it was shown that SUV39H1 and SNAI1 were concomitantly upregulated in MCF10A cells following TGFβ1 induction. These factors have been shown to interact with each other establishing a repressed state of the *CDH1* promoter by increasing the levels of H3K9me3 [115].

ZEB1 and ZEB2 are key factors regulating CDH1 expression and their connection with EMT and metastasis of cancer cells has been well established [121]. In a recent study it was shown that the ZEB1 promoter may exist in a poised state containing both marks of activation (H3K4me3) and repression (H3K27me3) [122]. In epithelial cells, ZEB1 is not expressed due to the bivalent marks on its promoter, whereas TGFβ1-mediated EMT induction resulted in removal of H3K27me3 from the ZEB1 promoter thereby facilitating its expression.

Similarly, removal of H3K27me3 by KDM6B was also shown to be essential for the induction of *SNAI1* expression during TGFβ1-induced EMT [104].

Apart from histone methylation, histone acetylation on the genes encoding EMT transcription factors has also been investigated. In this regard, it was previously shown that histone deacetylases (HDACs) modulated the chromatin state upon stimulus of extracellular signals like hypoxia [46]. Upon hypoxia, a well-described inducer of cancer cell aggressiveness and EMT, HDAC3 was recruited to epithelial genes such as *CDH1* leading to decreased H3K4ac, a subsequent increase in H3K4me2 and H3K27me3, and ultimately gene repression. On the other hand, mesenchymal genes such as Vimentin showed decreased H3K4ac, increased H3K4me2 but decreased H3K27me3. Here, HDAC3 interacted with WDR5, leading to methylation of H3K4 in hypoxic cells.

**Figure 4:**
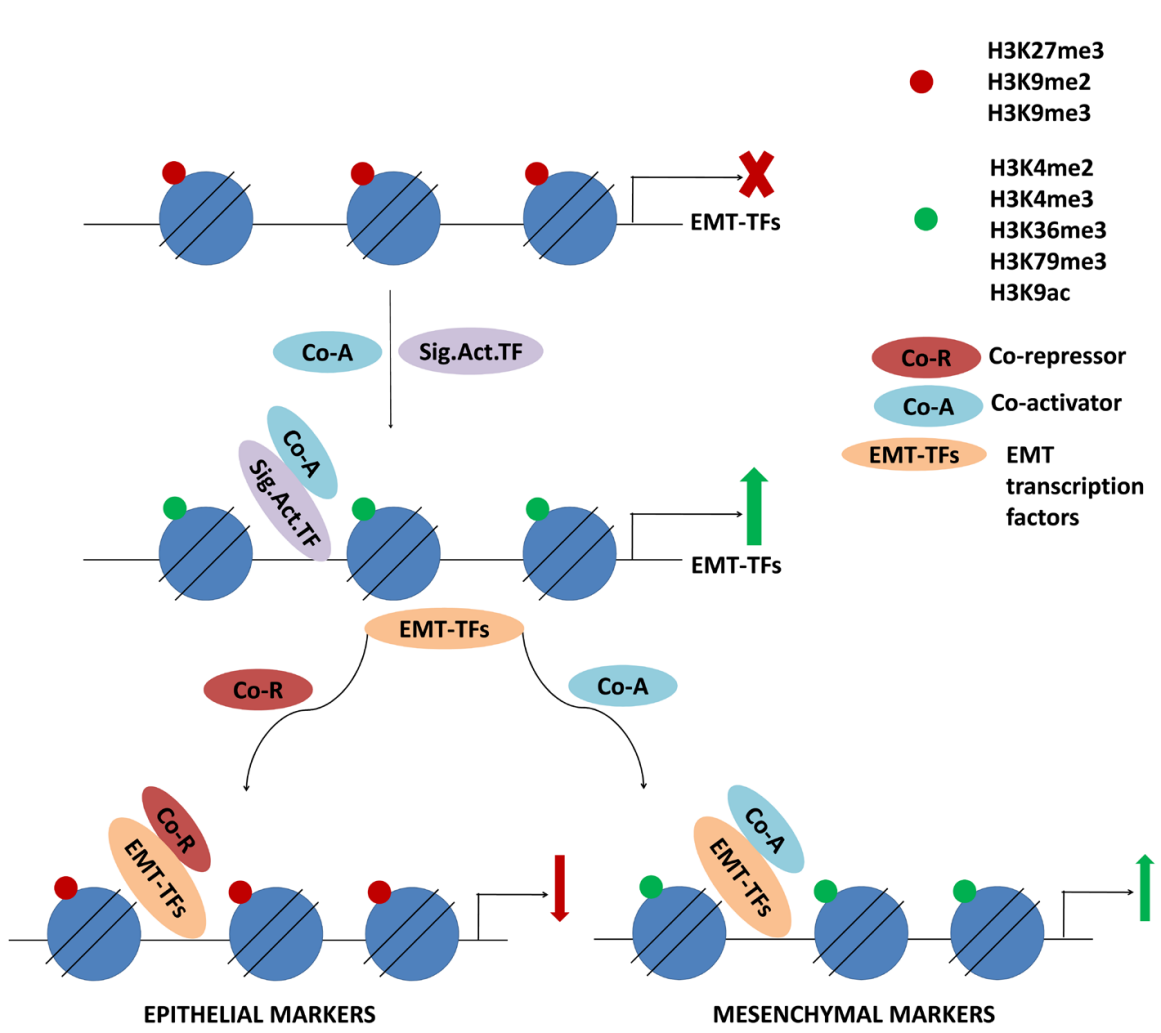
Signaling activators induce transcription of EMT-TFs which in turn regulate histone modifications on target genes Upon inducing signal, activating histone modifications on the genes of EMT-TFs promote transcription. EMT-TFs then interact with epigenetic regulators to mark the target genes for activation or repression.

In non-small cell lung cancer cell lines it was also shown that ZEB1 downregulated its target genes, e.g. EpCAM (epithelial cell adhesion molecule) by globally decreasing H3K27ac marks on these genes [123]. These findings are critical in understanding that the epigenetic regulation occurs relatively upstream of these markers or transcription factors, which may lead to the evaluation of these upstream EMT regulators as potential targets in anti-metastatic therapy. Figure 4 demonstrates the cascade of epigenetic events that control the transcriptional regulation of EMT transcription factors in response to EMT stimuli. The EMT TFs further function to directly regulate the transcription of epithelial and mesenchymal genes together with epigenetic coregulators.

### Perspectives

Cancer cells exploit epigenetic regulatory mechanisms for increased survival and resistance to apoptosis. It is now well established that chromatin regulation is an important determinant in controlling EMT, MET and cancer progression [119]. As the role of epigenetics in cancer becomes more apparent, we envisage that new strategies for the treatment of cancer will be developed that specifically focus on epigenetic dysregulation found in cancer cells. Targeting the molecular mechanisms for the methylation or acetylation of histones as shown partly in hematological malignancies could be a potential strategy of preventing EMT from occurring in solid tumors, thus interfering with local and systemic tissue infiltration. Several studies have now established various inhibitors targeting the key enzymes regulating the histone modifications that could potentially prevent the onset of EMT and tumor progression. For example, histone deacetylase inhibitors have been characterized that work on various HDACs, thereby maintaining the fine balance between HATs and HDACs [124]. EZH2, a well-known HMT implicated in regulating EMT by affecting H3K27me3 on EMT target genes, can be inhibited using the newly developed inhibitor EPZ005687. As a proof of principle, EPZ005687 was found to reduce H3K27me3 in lymphoma cells and render them more susceptible to induction of apoptosis [125]. Additionally, histone demethylases such as KDM6B/JMJD3 contribute to EMT by removing repressive marks from genes encoding EMT-TFs. A specific inhibitor for these demethylases such as the newly developed KDM6A/B inhibitor GSK-J4 was able to prevent the upregulation of EMT genes like SNAI1 [126]. JMJD3 has been shown to activate INK4a/ARF locus by its demethylase activity [127]. Hence, inhibition of JMJD3 might provide a viable approach for limiting epigenetic plasticity during tumor progression.

An exception to the family of SET domain containing methyltransferases is DOT1L which is responsible for H3K79 methylation. In AML with recurrent mixed lineage leukemia (MLL) gene translocation resulting in aggressive leukemias, DOT1L is aberrantly recruited to MLL target genes via interaction with the MLL translocation product where the expression of these genes also requires DOT1L histone methyltransferase activity [128]. In addition, DOT1L was shown to play a central role in the regulation of Wnt/β-catenin-regulated gene transcription [129,130]. Given the importance of the Wnt pathway in controlling EMT, it is possible that targeting DOT1L, for example via the small molecule inhibitor EPZ004777 which prevents H3K79 methylation on leukemic genes and selectively targets the cell carrying the MLL translocation [131], may represent a potential therapeutic target for anti-EMT therapy in specific cases where Wnt signaling plays a central role.

As we continue to elucidate the molecular circuits of epithelial-mesenchymal plasticity and its consequences for different aspects of tumor progression, we are obtaining a more precise view of the local and global epigenetic alterations in cancer cells undergoing EMT. We are just now beginning to understand how those changes induce EMT and MET or function to maintain epithelial and mesenchymal phenotypes. In sharp contrast to transcription factors, epigenetic regulators are amenable to targeting by small molecule inhibitors. Therefore, specific approaches targeting them may yield newer and better treatments based on characterized molecular mechanisms. It remains to be seen whether single inhibitors of EMT regulators will demonstrate clinical utility with regard to cancer invasion and metastasis prevention. A better understanding of the epigenetic mechanisms controlling the EMT and MET processes during distinct steps of metastasis formation together with the development of new, highly specific small molecule inhibitors will be essential for testing the feasibility of this approach and taking the next step toward individualized epigenetic tumor therapy.

## References

[1] Katalinic A (2010). The burden of cancer-actual and future developments. Tumor Biology.

[2] Valastyan S, Weinberg RA (2011). Review Tumor Metastasis :. Molecular Insights and Evolving Paradigms.

[3] Boutet A, De Frutos CA, Maxwell PH, Mayol MJ, Romero J, Nieto MA (2006). Snail activation disrupts tissue homeostasis and induces fibrosis in the adult kidney. EMBO J. 2006/11/10 ed.

[4] Nieto MA (2011). The Ins and Outs of the Epithelial to Mesenchymal Transition in Health and Disease. Annu Rev Cell Dev Biol. 2011/07/12 ed.

[5] Yang J, Weinberg RA (2008). Review Epithelial-Mesenchymal Transition :. At the Crossroads of Development and Tumor Metastasis.

[6] Scheel C, Weinberg RA (2012). Cancer stem cells and epithelial-mesenchymal transition: Concepts and molecular links. Semin Cancer Biol. 2012/05/05 ed.

[7] Ell B, Kang Y, Elsevier Ltd (2013). Transcriptional control of cancer metastasis. Trends in cell biology.

[8] Mani SA, Guo W, Liao MJ, Eaton EN, Ayyanan A, Zhou AY (2008). The epithelial-mesenchymal transition generates cells with properties of stem cells. Cell.

[9] Morel AP, Lievre M, Thomas C, Hinkal G, Ansieau S, Puisieux A (2008). Generation of breast cancer stem cells through epithelial-mesenchymal transition. PLoS One.

[10] Tsai JH, Donaher JL, Murphy DA, Chau S, Yang J, Elsevier Inc (2012). Article Spatiotemporal Regulation of Epithelial-Mesenchymal Transition Is Essential for Squamous Cell Carcinoma Metastasis. Cancer Cell.

[11] Chaffer CL, Brennan JP, Slavin JL, Blick T, Thompson EW, Williams ED (2006). Mesenchymal-to-epithelial transition facilitates bladder cancer metastasis: role of fibroblast growth factor receptor-2. Cancer Res.

[12] Kowalski PJ, Rubin MA, Kleer CG (2003). E-cadherin expression in primary carcinomas of the breast and its distant metastases. Breast cancer research : BCR.

[13] Yu M, Bardia A, Wittner BS, Stott SL, Smas ME, Ting DT (2013). Circulating breast tumor cells exhibit dynamic changes in epithelial and mesenchymal composition. Science.

[14] Wang Y, Shang Y, Elsevier (2013). Epigenetic control of epithelial-to-mesenchymal transition and cancer metastasis. Experimental Cell Research.

[15] Dupont C, Armant DR, Brenner CA (2009). Epigenetics: definition, mechanisms and clinical perspective. Semin Reprod Med.

[16] Lachner M, Jenuwein T (2002). The many faces of histone lysine methylation. Curr Opin Cell Biol.

[17] Loyola A, Almouzni G (2004). Histone chaperones, a supporting role in the limelight. Biochimica et Biophysica Acta (BBA) - Gene Structure and Expression.

[18] Peterson CL, Tamkun JW (1995). The SWI-SNF complex: a chromatin remodeling machine?. Trends in Biochemical Sciences.

[19] Hawkins RD, Hon GC, Lee LK, Ngo Q, Lister R, Pelizzola M (2010). Distinct epigenomic landscapes of pluripotent and lineage-committed human cells. Cell Stem Cell.

[20] Jones PA, Baylin SB (2007). The epigenomics of cancer. Cell.

[21] Jenuwein T, Allis CD (2001). Translating the histone code. Science.

[22] Kouzarides T (2007). Chromatin modifications and their function. Cell.

[23] James LI, Frye SV (2013). Targeting Chromatin Readers. Clin Pharmacol Ther.

[24] You JS, Jones PA (2012). Review Cancer Genetics and Epigenetics :. Two Sides of the Same Coin?.

[25] Thiery JP, Acloque H, Huang RY, Nieto MA (2009). Epithelial-mesenchymal transitions in development and disease. Cell.

[26] Cedar H, Bergman Y (2009). Linking DNA methylation and histone modification: patterns and paradigms. Nat Rev Genet.

[27] Campos EI, Reinberg D (2009). Histones: annotating chromatin. Annu Rev Genet.

[28] McCabe MT, Brandes JC, Vertino PM (2009). Cancer DNA methylation: molecular mechanisms and clinical implications. Clin Cancer Res.

[29] Baylin SB, Esteller M, Rountree MR, Bachman KE, Schuebel K, Herman JG (2001). Aberrant patterns of DNA methylation, chromatin formation and gene expression in cancer. Hum Mol Genet.

[30] Lombaerts M, van Wezel T, Philippo K, Dierssen JWF, Zimmerman RME, Oosting J (2006). E-cadherin transcriptional downregulation by promoter methylation but not mutation is related to epithelial-to-mesenchymal transition in breast cancer cell lines. Br J Cancer.

[31] Dumont N, Wilson MB, Crawford YG, Reynolds PA, Sigaroudinia M, Tlsty TD (2008). Sustained induction of epithelial to mesenchymal transition activates DNA methylation of genes silenced in basal-like breast cancers. Proc Natl Acad Sci USA.

[32] Hsu C-H, Peng K-L, Kang M-L, Chen Y-R, Yang Y-C, Tsai C-H (2012). TET1 suppresses cancer invasion by activating the tissue inhibitors of metalloproteinases. Cell Rep.

[33] Huang H, Jiang X, Li Z, Li Y, Song C-X, He C (2013). TET1 plays an essential oncogenic role in MLL-rearranged leukemia. Proc Natl Acad Sci USA.

[34] Yang H, Liu Y, Bai F, Zhang J-Y, Ma S-H, Liu J (2013). Tumor development is associated with decrease of TET gene expression and 5-methylcytosine hydroxylation. Oncogene.

[35] Song SJ, Ito K, Ala U, Kats L, Webster K, Sun SM (2013). The Oncogenic MicroRNA miR-22 Targets the TET2 Tumor Suppressor to Promote Hematopoietic Stem Cell Self-Renewal and Transformation. Cell Stem Cell.

[36] Grunstein M (1997). Histone acetylation in chromatin structure and transcription. Nature.

[37] Imhof A, Yang X-J, Ogryzko VV, Nakatani Y, Wolffe AP, Ge H (1997). Acetylation of general transcription factors by histone acetyltransferases. Current Biology.

[38] Peña C, García JM, García V, Silva J, Domínguez G, Rodríguez R (2006). The expression levels of the transcriptional regulators p300 and CtBP modulate the correlations between SNAIL, ZEB1, E-cadherin and vitamin D receptor in human colon carcinomas. International Journal of Cancer.

[39] Krubasik D, Iyer NG, English WR, Ahmed AA, Vias M, Roskelley C (2006). Absence of p300 induces cellular phenotypic changes characteristic of epithelial to mesenchyme transition. Br J Cancer.

[40] Jafarnejad SM, Li G (2012). Regulation of p53 by ING family members in suppression of tumor initiation and progression. Cancer Metastasis Rev.

[41] Qin L, Liu Z, Chen H, Xu J (2009). The Steroid Receptor Coactivator-1 Regulates Twist Expression and Promotes Breast Cancer Metastasis. Cancer Res.

[42] Agoulnik IU, Vaid A, Bingman WE, Erdeme H, Frolov A, Smith CL (2005). Role of SRC-1 in the Promotion of Prostate Cancer Cell Growth and Tumor Progression. Cancer Res.

[43] Zhou H-J, Yan J, Luo W, Ayala G, Lin S-H, Erdem H (2005). SRC-3 Is Required for Prostate Cancer Cell Proliferation and Survival. Cancer Res.

[44] Lydon JP, O'Malley BW (2011). Minireview: Steroid Receptor Coactivator-3: A Multifarious Coregulator in Mammary Gland Metastasis. Endocrinology.

[45] Miller T, Krogan NJ, Dover J, Erdjument-Bromage H, Tempst P, Johnston M (2001). COMPASS: A complex of proteins associated with a trithorax-related SET domain protein. PNAS.

[46] Wu M-Z, Tsai Y-P, Yang M-H, Huang C-H, Chang S-Y, Chang C-C (2011). Interplay between HDAC3 and WDR5 Is Essential for Hypoxia-Induced Epithelial-Mesenchymal Transition. Molecular Cell.

[47] Ringrose L, Paro R (2004). Epigenetic regulation of cellular memory by the Polycomb and Trithorax group proteins. Annu Rev Genet.

[48] Orlando V (2003). Polycomb, epigenomes, and control of cell identity. Cell.

[49] Hershko A, Ciechanover A (1998). The Ubiquitin System. Annual Review of Biochemistry.

[50] Xiao T, Kao C-F, Krogan NJ, Sun Z-W, Greenblatt JF, Osley MA (2005). Histone H2B Ubiquitylation Is Associated with Elongating RNA Polymerase II. Mol Cell Biol.

[51] Shukla A, Stanojevic N, Duan Z, Shadle T, Bhaumik SR (2006). Functional analysis of H2B-Lys-123 ubiquitination in regulation of H3-Lys-4 methylation and recruitment of RNA polymerase II at the coding sequences of several active genes in vivo. J Biol Chem.

[52] Minsky N, Shema E, Field Y, Schuster M, Segal E, Oren M (2008). Monoubiquitinated H2B is associated with the transcribed region of highly expressed genes in human cells. Nat Cell Biol.

[53] Wang H, Wang L, Erdjument-Bromage H, Vidal M, Tempst P, Jones RS (2004). Role of histone H2A ubiquitination in Polycomb silencing. Nature.

[54] Cao R, Tsukada Y, Zhang Y (2005). Role of Bmi-1 and Ring1A in H2A Ubiquitylation and Hox Gene Silencing. Molecular Cell.

[55] Zhu B, Zheng Y, Pham A-D, Mandal SS, Erdjument-Bromage H, Tempst P (2005). Monoubiquitination of Human Histone H2B: The Factors Involved and Their Roles in HOX Gene Regulation. Molecular Cell.

[56] Johnsen SA (2012). The enigmatic role of H2Bub1 in cancer. FEBS Letters. Federation of European Biochemical Societies.

[57] Shema E, Tirosh I, Aylon Y, Huang J, Ye C, Moskovits N (2008). The histone H2B-specific ubiquitin ligase RNF20/hBRE1 acts as a putative tumor suppressor through selective regulation of gene expression. Genes Dev.

[58] Prenzel T, Begus-Nahrmann Y, Kramer F, Hennion M, Hsu C, Gorsler T (2011). Estrogen-dependent gene transcription in human breast cancer cells relies upon proteasome-dependent monoubiquitination of histone H2B. Cancer Res.

[59] Song L-B, Li J, Liao W-T, Feng Y, Yu C-P, Hu L-J (2009). The polycomb group protein Bmi-1 represses the tumor suppressor PTEN and induces epithelial-mesenchymal transition in human nasopharyngeal epithelial cells. J Clin Invest.

[60] Yang M-H, Hsu DS-S, Wang H-W, Wang H-J, Lan H-Y, Yang W-H (2010). Bmi1 is essential in Twist1-induced epithelial-mesenchymal transition. Nat Cell Biol.

[61] Wellner U, Schubert J, Burk UC, Schmalhofer O, Zhu F, Sonntag A (2009). The EMT-activator ZEB1 promotes tumorigenicity by repressing stemness-inhibiting microRNAs. Nature Cell Biology. Nature Publishing Group.

[62] De la Cruz X, Lois S, Sánchez-Molina S, Martínez-Balbás MA (2005). Do protein motifs read the histone code?. BioEssays.

[63] Haynes SR, Dollard C, Winston F, Beck S, Trowsdale J, Dawid IB (1992). The bromodomain: a conserved sequence found in human, Drosophila and yeast proteins. Nucl Acids Res.

[64] Jeanmougin F, Wurtz J-M, Le Douarin B, Chambon P, Losson R (1997). The bromodomain revisited. Trends in Biochemical Sciences.

[65] Florence B, Faller DV (2001). You bet-cha: a novel family of transcriptional regulators. Front Biosci.

[66] Wu S-Y, Chiang C-M (2007). The double bromodomain-containing chromatin adaptor Brd4 and transcriptional regulation. J Biol Chem.

[67] Zuber J, Shi J, Wang E, Rappaport AR, Herrmann H, Sison EA (2011). RNAi screen identifies Brd4 as a therapeutic target in acute myeloid leukaemia. Nature.

[68] French CA, Miyoshi I, Kubonishi I, Grier HE, Perez-Atayde AR, Fletcher JA (2003). BRD4-NUT Fusion Oncogene A Novel Mechanism in Aggressive Carcinoma. Cancer Res.

[69] French CA, Kutok JL, Faquin WC, Toretsky JA, Antonescu CR, Griffin CA (2004). Midline Carcinoma of Children and Young Adults With NUT Rearrangement. JCO.

[70] French CA, Ramirez CL, Kolmakova J, Hickman TT, Cameron MJ, Thyne ME (2007). BRD–NUT oncoproteins: a family of closely related nuclear proteins that block epithelial differentiation and maintain the growth of carcinoma cells. Oncogene.

[71] Alsarraj J, Walker RC, Webster JD, Geiger TR, Crawford NPS, Simpson RM (2011). Deletion of the Proline-Rich Region of the Murine Metastasis Susceptibility Gene Brd4 Promotes Epithelial-to-Mesenchymal Transition- and Stem Cell-Like Conversion. Cancer Res.

[72] Filippakopoulos P, Qi J, Picaud S, Shen Y, Smith WB, Fedorov O (2010). Selective inhibition of BET bromodomains. Nature.

[73] Delmore JE, Issa GC, Lemieux ME, Rahl PB, Shi J, Jacobs HM (2011). BET Bromodomain Inhibition as a Therapeutic Strategy to Target c-Myc. Cell.

[74] Mertz JA, Conery AR, Bryant BM, Sandy P, Balasubramanian S, Mele DA (2011). Targeting MYC dependence in cancer by inhibiting BET bromodomains. PNAS.

[75] Kim J, Daniel J, Espejo A, Lake A, Krishna M, Xia L (2006). Tudor, MBT and chromo domains gauge the degree of lysine methylation. EMBO Rep.

[76] Tang M, Shen H, Jin Y, Lin T, Cai Q, Pinard MA (2013). The Malignant Brain Tumor (MBT) Domain Protein SFMBT1 Is an Integral Histone Reader Subunit of the LSD1 Demethylase Complex for Chromatin Association and Epithelial-to-mesenchymal Transition. J Biol Chem.

[77] Yap KL, Zhou M-M (2011). Structure and Mechanisms of Lysine Methylation Recognition by the Chromodomain in Gene Transcription. Biochemistry.

[78] Levine SS, Weiss A, Erdjument-Bromage H, Shao Z, Tempst P, Kingston RE (2002). The Core of the Polycomb Repressive Complex Is Compositionally and Functionally Conserved in Flies and Humans. Mol Cell Biol.

[79] Francis NJ, Kingston RE, Woodcock CL (2004). Chromatin Compaction by a Polycomb Group Protein Complex. Science.

[80] Long J, Zuo D, Park M (2005). Pc2-mediated sumoylation of Smad-interacting protein 1 attenuates transcriptional repression of E-cadherin. J Biol Chem.

[81] Vandewalle C, Comijn J, De Craene B, Vermassen P, Bruyneel E, Andersen H (2005). SIP1/ZEB2 induces EMT by repressing genes of different epithelial cell-cell junctions. Nucleic Acids Res.

[82] Kokura K, Sun L, Bedford MT, Fang J (2010). Methyl-H3K9-binding protein MPP8 mediates E-cadherin gene silencing and promotes tumour cell motility and invasion. EMBO J.

[83] Sif S (2004). ATP-dependent nucleosome remodeling complexes: Enzymes tailored to deal with chromatin. Journal of Cellular Biochemistry.

[84] Avvakumov N, Nourani A, Côtô J (2011). Histone Chaperones: Modulators of Chromatin Marks. Molecular Cell.

[85] Wong AKC, Shanahan F, Chen Y, Lian L, Ha P, Hendricks K (2000). BRG1, a Component of the SWI-SNF Complex, Is Mutated in Multiple Human Tumor Cell Lines. Cancer Res.

[86] Barker N, Hurlstone A, Musisi H, Miles A, Bienz M, Clevers H (2001). The chromatin remodelling factor Brg-1 interacts with β-catenin to promote target gene activation. EMBO J.

[87] Sánchez-Tilló E, Lázaro a, Torrent R, Cuatrecasas M, Vaquero EC, Castells a (2010). ZEB1 represses E-cadherin and induces an EMT by recruiting the SWI/SNF chromatin-remodeling protein BRG1. Oncogene.

[88] Fujita N, Jaye DL, Kajita M, Geigerman C, Moreno CS, Wade PA (2003). MTA3, a Mi-2/NuRD Complex Subunit, Regulates an Invasive Growth Pathway in Breast Cancer. Cell.

[89] Bedi U, Scheel AH, Hennion M, Begus-Nahrmann Y, Rüschoff J, Johnsen SA (2014). SUPT6H controls estrogen receptor activity and cellular differentiation by multiple epigenomic mechanisms. Oncogene [Internet].

[90] Koman IE, Commane M, Paszkiewicz G, Hoonjan B, Pal S, Safina A (2012). Targeting FACT Complex Suppresses Mammary Tumorigenesis in Her2/neu Transgenic Mice. Cancer Prev Res.

[91] Kari V, Shchebet A, Neumann H, Johnsen SA (2011). The H2B ubiquitin ligase RNF40 cooperates with SUPT16H to induce dynamic changes in chromatin structure during DNA double-strand break repair. Cell Cycle.

[92] Barretina J, Caponigro G, Stransky N, Venkatesan K, Margolin AA, Kim S (2012). The Cancer Cell Line Encyclopedia enables predictive modelling of anticancer drug sensitivity. Nature.

[93] Môtivier R, Gallais R, Tiffoche C, Le Pôron C, Jurkowska RZ, Carmouche RP (2008). Cyclical DNA methylation of a transcriptionally active promoter. Nature.

[94] Kangaspeska S, Stride B, Môtivier R, Polycarpou-Schwarz M, Ibberson D, Carmouche RP (2008). Transient cyclical methylation of promoter DNA. Nature.

[95] Môtivier R, Penot G, Hübner MR, Reid G, Brand H, Kos M (2003). Estrogen receptor-alpha directs ordered, cyclical, and combinatorial recruitment of cofactors on a natural target promoter. Cell.

[96] Arrowsmith CH, Bountra C, Fish PV, Lee K, Schapira M (2012). Epigenetic protein families: a new frontier for drug discovery. Nat Rev Drug Discov.

[97] Lei W, Zhang K, Pan X, Hu Y, Wang D, Yuan X (2010). Histone deacetylase 1 is required for transforming growth factor-β1-induced epithelial–mesenchymal transition. The International Journal of Biochemistry & Cell Biology.

[98] Yoshikawa M, Hishikawa K, Marumo T, Fujita T (2007). Inhibition of Histone Deacetylase Activity Suppresses Epithelial-to-Mesenchymal Transition Induced by TGF-β1 in Human Renal Epithelial Cells. JASN.

[99] Byles V, Zhu L, Lovaas JD, Chmilewski LK, Wang J, Faller DV (2012). SIRT1 induces EMT by cooperating with EMT transcription factors and enhances prostate cancer cell migration and metastasis. Oncogene.

[100] Shi Y, Lan F, Matson C, Mulligan P, Whetstine JR, Cole PA (2004). Histone demethylation mediated by the nuclear amine oxidase homolog LSD1. Cell.

[101] Lin T, Ponn A, Hu X, Law BK, Lu J (2010). Requirement of the histone demethylase LSD1 in Snai1-mediated transcriptional repression during epithelial-mesenchymal transition. Oncogene.

[102] Ferrari-Amorotti G, Fragliasso V, Esteki R, Prudente Z, Soliera AR, Cattelani S (2013). Inhibiting Interactions of Lysine Demethylase LSD1 with Snail/Slug Blocks Cancer Cell Invasion. Cancer Res.

[103] Amente S, Lania L, Majello B (2013). The histone LSD1 demethylase in stemness and cancer transcription programs. Biochimica et Biophysica Acta (BBA) - Gene Regulatory Mechanisms.

[104] Ramadoss S, Chen X, Wang C-Y (2012). Histone demethylase KDM6B promotes epithelial-mesenchymal transition. J Biol Chem.

[105] Zhao L, Li W, Zang W, Liu Z, Xu X, Yu H (2013). JMJD2B promotes epithelial-mesenchymal transition by cooperating with β-catenin and enhances gastric cancer metastasis. Clin Cancer Res.

[106] Zhang X-Y, Varthi M, Sykes SM, Phillips C, Warzecha C, Zhu W (2008). The Putative Cancer Stem Cell Marker USP22 Is a Subunit of the Human SAGA Complex Required for Activated Transcription and Cell-Cycle Progression. Molecular Cell.

[107] Liu Y-L, Jiang S-X, Yang Y-M, Xu H, Liu J-L, Wang X-S (2012). USP22 Acts as an Oncogene by the Activation of BMI-1-Mediated INK4a/ARF Pathway and Akt Pathway. Cell Biochem Biophys.

[108] Glinsky GV, Berezovska O, Glinskii AB (2005). Microarray analysis identifies a death-from-cancer signature predicting therapy failure in patients with multiple types of cancer. Journal of Clinical Investigation.

[109] Liu Y-L, Yang Y-M, Xu H, Dong X-S (2011). Aberrant expression of USP22 is associated with liver metastasis and poor prognosis of colorectal cancer. Journal of Surgical Oncology.

[110] Nicassio F, Corrado N, Vissers JHA, Areces LB, Bergink S, Marteijn JA (2007). Human USP3 is a chromatin modifier required for S phase progression and genome stability. Curr Biol.

[111] Buus R, Faronato M, Hammond DE, Urbô S, Clague MJ (2009). Deubiquitinase Activities Required for Hepatocyte Growth Factor-Induced Scattering of Epithelial Cells. Current Biology.

[112] Tepass U, Truong K, Godt D, Ikura M, Peifer M (2000). Cadherins in embryonic and neural morphogenesis. Nat Rev Mol Cell Biol.

[113] Birchmeier W, Behrens J (1994). Cadherin expression in carcinomas: role in the formation of cell junctions and the prevention of invasiveness. Biochim Biophys Acta.

[114] Batlle E, Sancho E, Francí C, Domínguez D, Monfar M, Baulida J (2000). The transcription factor snail is a repressor of E-cadherin gene expression in epithelial tumour cells. Nat Cell Biol.

[115] Dong C, Wu Y, Wang Y, Wang C, Kang T, Rychahou PG (2013). Interaction with Suv39H1 is critical for Snail-mediated E-cadherin repression in breast cancer. Oncogene.

[116] Dong C, Wu Y, Yao J, Wang Y, Yu Y, Rychahou G (2012). G9a interacts with Snail and is critical for Snail-mediated E-cadherin repression in human breast cancer. J Clin Invest.

[117] Herranz N, Pasini D, Díaz VM, Francí C, Gutierrez A, Dave N (2008). Polycomb complex 2 is required for E-cadherin repression by the Snail1 transcription factor. Mol Cell Biol.

[118] Lin Y, Wu Y, Li J, Dong C, Ye X, Chi Y-I (2010). The SNAG domain of Snail1 functions as a molecular hook for recruiting lysine-specific demethylase 1. EMBO J.

[119] Tam WL, Weinberg RA (2013). The epigenetics of epithelial-mesenchymal plasticity in cancer. Nat Med.

[120] Liu P, Ramachandran S, Ali Seyed M, Scharer CD, Laycock N, Dalton WB (2006). Sex-determining region Y box 4 is a transforming oncogene in human prostate cancer cells. Cancer Res.

[121] Tiwari N, Tiwari VK, Waldmeier L, Balwierz PJ, Arnold P, Pachkov M (2013). Sox4 is a master regulator of epithelial-mesenchymal transition by controlling Ezh2 expression and epigenetic reprogramming. Cancer cell.

[122] Spaderna S, Schmalhofer O, Wahlbuhl M, Dimmler A, Bauer K, Sultan A (2008). The transcriptional repressor ZEB1 promotes metastasis and loss of cell polarity in cancer. Cancer Res.

[123] Chaffer CL, Marjanovic ND, Lee T, Bell G, Kleer CG, Reinhardt F (2013). Poised chromatin at the ZEB1 promoter enables breast cancer cell plasticity and enhances tumorigenicity. Cell.

[124] Roche J, Nasarre P, Gemmill R, Baldys A, Pontis J, Korch C (2013). Global Decrease of Histone H3K27 Acetylation in ZEB1-Induced Epithelial to Mesenchymal Transition in Lung Cancer Cells. Cancers.

[125] Minucci S, Pelicci PG (2006). Histone deacetylase inhibitors and the promise of epigenetic (and more) treatments for cancer. Nat Rev Cancer.

[126] Knutson SK, Wigle TJ, Warholic NM, Sneeringer CJ, Allain CJ, Klaus CR (2012). A selective inhibitor of EZH2 blocks H3K27 methylation and kills mutant lymphoma cells. Nat Chem Biol.

[127] Kruidenier L, Chung C, Cheng Z, Liddle J, Che K, Joberty G (2012). A selective jumonji H3K27 demethylase inhibitor modulates the proinflammatory macrophage response. Nature.

[128] Agger K, Cloos PAC, Rudkjær L, Williams K, Andersen G, Christensen J (2009). The H3K27me3 demethylase JMJD3 contributes to the activation of the INK4A–ARF locus in response to oncogene- and stress-induced senescence. Genes Dev.

[129] Bernt KM, Zhu N, Sinha AU, Vempati S, Faber J, Krivtsov AV (2011). MLL-Rearranged Leukemia Is Dependent on Aberrant H3K79 Methylation by DOT1L. Cancer Cell.

[130] Mahmoudi T, Boj SF, Hatzis P, Li VSW, Taouatas N, Vries RGJ (2010). The Leukemia-Associated Mllt10/Af10-Dot1l Are Tcf4/β-Catenin Coactivators Essential for Intestinal Homeostasis. PLoS Biol.

[131] Mohan M, Herz H-M, Takahashi Y-H, Lin C, Lai KC, Zhang Y (2010). Linking H3K79 trimethylation to Wnt signaling through a novel Dot1-containing complex (DotCom). Genes Dev.

[132] Daigle SR, Olhava EJ, Therkelsen CA, Majer CR, Sneeringer CJ, Song J (2011). Selective Killing of Mixed Lineage Leukemia Cells by a Potent Small-Molecule DOT1L Inhibitor. Cancer Cell.

[133] Peinado H, Ballestar E, Esteller M, Cano A (2004). Snail Mediates E-Cadherin Repression by the Recruitment of the Sin3A/Histone Deacetylase 1 (HDAC1)/HDAC2 Complex. Mol Cell Biol.

[134] Barneda-Zahonero B, Parra M (2012). Histone deacetylases and cancer. Molecular Oncology.

[135] Rajasekhar VK, Begemann M (2007). Concise review: roles of polycomb group proteins in development and disease: a stem cell perspective. Stem Cells.

[136] Pasini D, Bracken AP, Jensen MR, Lazzerini Denchi E, Helin K (2004). Suz12 is essential for mouse development and for EZH2 histone methyltransferase activity. EMBO J.

[137] Haupt Y, Alexander WS, Barri G, Peter Klinken S, Adams JM (1991). Novel zinc finger gene implicated as myc collaborator by retrovirally accelerated lymphomagenesis in Eμ-myc transgenic mice. Cell.

[138] Yang M-H, Hsu DS-S, Wang H-W, Wang H-J, Lan H-Y, Yang W-H (2010). Bmi1 is essential in Twist1-induced epithelial-mesenchymal transition. Nat Cell Biol.

[139] Yu C-C, Lo W-L, Chen Y-W, Huang P-I, Hsu H-S, Tseng L-M (2010). Bmi-1 Regulates Snail Expression and Promotes Metastasis Ability in Head and Neck Squamous Cancer-Derived ALDH1 Positive Cells. Journal of Oncology [Internet].

[140] Wang H, Wang L, Erdjument-Bromage H, Vidal M, Tempst P, Jones RS (2004). Role of histone H2A ubiquitination in Polycomb silencing. Nature.

[141] Lin T, Ponn A, Hu X, Law BK, Lu J (2010). Requirement of the histone demethylase LSD1 in Snai1-mediated transcriptional repression during epithelial-mesenchymal transition. Oncogene.

[142] McDonald OG, Wu H, Timp W, Doi A, Feinberg AP (2011). Genome-scale epigenetic reprogramming during epithelial-to-mesenchymal transition. Nat Struct Mol Biol.

